# Supplementary material for: An evidence-based approach to assessing the effectiveness of training regimen on athlete performance: Youth soccer as a case study
Source: PLoS One. 2022 Nov 1;17(11):e0276762. doi: 10.1371/journal.pone.0276762 (PMC9624410; doi:10.1371/journal.pone.0276762)
Supplement: S1 Appendix — (DOCX) [file pone.0276762.s001.docx]

**Appendix: Akaike’s Information Criterion (AIC), Akaike Weights and Multimodel Inference**

AIC provides an approach to model selection that seeks a parsimonious model that adequately describes the data (Akaike 1973, Burnham and Anderson 2002). AIC is computed as follows:

AIC = -2 ln (*L*) + 2*q*,

where ln (*L*) is the natural logarithm of the likelihood function (evaluated at the maximum likelihood estimates) and *q* is the number of parameters in the model. The first term reflects the degree of fit of the model to the data, whereas the latter term is sometimes viewed as a penalty for the number of model parameters.

Under a model selection paradigm, AIC is computed for each model considered in the *a priori* candidate model set. The relative values of AIC are of interest, with a focus on ΔAIC*_j_*, the difference between the AIC value for model *j*, AIC*_j_*, and the smallest AIC*_k_* value for all models in the model set, AIC*_min_*:

ΔAIC*_j_* = AIC*_j_* - AIC*_min_* .

The model(s) with the lower ΔAIC*_j_* are the “better” models in the set, in the sense of providing parsimonious descriptions of the data.

Akaike weights, *w_j_*, are computed based on exp (- ΔAIC*_j_* /2), which can be viewed as the relative likelihood of model *j*, given the data and the other members of the model set. Specifically, Akaike weights are computed by normalizing these relative likelihood values to unity:

$w_{j}= \frac{exp(\frac{{-\Delta AIC}_{j}}{2})}{\sum_{j=1}^{J} exp(\frac{{-\Delta AIC}_{j}}{2})}$ ,

where *J* is the number of models in the candidate set (Burnham and Anderson 2002).

Akaike weights can be used to obtain model-averaged estimates of focal model parameters:

$\hat{\theta^{*}}=\sum_{j=1}^{J} w_{j}\hat{\theta_{j}}$ ,

where $\hat{\theta_{j}}$ denotes the maximum likelihood estimate of parameter $\theta$ under model *j*, and $\hat{\theta^{*}}$ denotes the model-averaged estimate (Buckland et al. 1997, Burnham and Anderson 2002). The variance of the model-averaged estimator can be estimated as:

$\hat{var} (\hat{\theta^{*}}$) = $\left[ \sum_{j=1}^{J} w_{j}\sqrt{{\hat{var} (\hat{\theta_{j}} | M_{j})+(\hat{\theta_{j}}- \hat{\theta^{*}})}^{2}} \right]^{2}$ ,

where $\hat{var} (\hat{\theta_{j}} | M_{j})$ is the variance associated with $\hat{\theta_{j}}$, conditional on model *M_j_* . The second additive term under the radical, ${(\hat{\theta_{j}}- \hat{\theta^{*}})}^{2}$, reflects the variance component associated with model uncertainty (Buckland et al. 1997, Burnham and Anderson 2002). Model-averaging was developed for use with model parameters but can also be used to compute derived parameters that do not appear directly in the model.

In the application of these methods to the specific analyses of this study, model-averaged predictions were computed for the relationship between predicted skill improvement and both training time and commitment score. Each model produced an estimate of improvement for each training time or commitment score based on the $\beta$estimates for that model and each possible value of $x_{i\Delta t}$. In the case of model *4*, predicted improvement also required $y_{it_{1}}$; hence there are *N_A_* -1 different instantiations of model *4*, one for each value of initial proficiency (2 athletes had identical initial proficiency values). Akaike model weights (Burnham and Anderson 2002, see above) based on the model selection statistics were used to compute weighted average improvement estimates, which were used to plot the model-averaged relationship between change in proficiency and training time. We used the median value of initial proficiency to compute the estimates under model *4* required for averaging.

We also computed model-averaged estimates of $\beta_{1}$ as weighted means of the $\hat{\beta}_{1}$. Model-averaging of $\hat{\beta}_{l}$parameter estimates is not generally recommended, as $\beta$parameters can attain different meanings in different models. However, $\beta_{1}$ has the same basic meaning in models *2-4*, as the increase in proficiency immediately following the first 16 hours of training (i.e., before any leveling off of gains as in model *3*). Model *1* does not have a $\beta_{1}$ parameter, implying that $\beta_{1}=0,$so 0 was used as the estimate associated with the Akaike weight for model *1*. Model-averaged estimates permit inference in the face of uncertainty about which model provides the best description of the processes generating the data. If one model is clearly much better supported than all others, then its weight will approach 1 and the model-averaged estimate or plot will correspond to this model. In the case of uncertainty, the model-averaged *E*($\hat{\beta}_{1})$, and the relationship that it defines, will appear as intermediate to those based on the single models.
