# Supplementary material for: An evidence-based approach to assessing the effectiveness of training regimen on athlete performance: Youth soccer as a case study
Source: PLoS One. 2022 Nov 1;17(11):e0276762. doi: 10.1371/journal.pone.0276762 (PMC9624410; doi:10.1371/journal.pone.0276762)
Supplement: Attachment — Submitted filename: Response to Editor (9-23-2022).docx [file pone.0276762.s005.docx]

Sept. 21, 2022

Rafael Franco Soares Oliveira
Academic Editor
PLOS ONE

Re: PONE-D-22-04804

An evidence-based approach to assessing the effectiveness of training regimen on athlete performance: youth soccer as a case study

Dear Dr. Oliviera:

Thank you for your invitation to submit a revised version of our manuscript. As requested in your message of September 19, 2022, in this letter we respond to your comments and recommendations. Below, we will copy your main comments and respond in italics.

“First an English revision/proofreading should be performed because there are several expressions and sentences not well written. Moreover, the work was written in the first person, but it should be changed to the third.”

*We have tried to improve and clarify the writing throughout, and we have rewritten the entire manuscript and appendix in the third person. As you can see in the Track Changes copy of our revision, virtually all paragraphs contain changes associated with these recommendations.*

“Discussion should start with the aims of the study and the main results. In addition, there should be a limitation, a clear practical application and conclusion sections for this paper.”

*We have rewritten portions of the Discussion in order to follow your suggestions. We added a new paragraph to begin the Discussion (lines 664-672) in which we stated study aims and results. The primary limitation of the study is that inferences are based on a single training program (see lines 761-763). Practical applications are both specific (coaches/trainers encouraging greater commitment; lines 763-768) and general (use of similar methodological approaches to promote evidence-based training; lines 768-770; more detail and recommendations in lines 642-656). A conclusions paragraph was added (lines 725-757).*

We tried to follow all of your recommendations and hope that our responses seem reasonable to you. We look forward to hearing from you, and we will try to address any remaining concerns you may have. Thank you for considering our manuscript.

Sincerely,

James D. Nichols
